# A System of Practical Medicine, Comprised in a Series of Original Dissertations

**Published:** 1841-05-08

**Authors:** 


### BIBLIOGRAPHICAL NOTICE.

*A System of Practical Medicine, comprised in a series of Original Dissertations.* Arranged and edited by Alexander Tweedie, M. D., F. R. S., Fellow of the Royal College of Physicians, &c. Vol. IV. Philadelphia: Lea & Blanchard, 1841.

*Diseases of Digestive and Uterine Organs.*

The fourth volume of this work includes the diseases of the arteries and veins, of the organs of digestion, of the urinary organs, and of the uterus and its appendages. The papers on the arteries and veins are from Dr. Joy, the author of those on the heart, which appeared in the third volume; those of the organs of digestion, chiefly from Dr. Symonds; of the urinary organs, from Dr. Christison; and of the uterine organs, from Drs. Ferguson and Simpson.

The diseases of the blood-vessels are less complex and perhaps less interesting than those of the central organ of the circulation. Dr. Joy has, however, developed all that is necessary to be known respecting them, but has, perhaps, laid too little stress upon aortitis as a cause of aneurism.

The description of the affections of the alimentary canal is extremely full, as far as it relates to diseases of the stomach and liver, especially of the latter organ. Colitis or dysentery is less fully treated of, because this disease occurs only in a mitigated form in the British islands, and does not assume the severe character of our warmer summer climate. The deficiency in this respect is supplied by additional matter.

The articles on the diseases of the kidneys constitute a concise but excellent monograph on the subject. This is one of the most finished series of articles in the five volumes, and, from the general reputation of the author, as well as his peculiar devotion to the study of these diseases, there is no portion which will be read and consulted with more advantage.

The uterine diseases are satisfactorily treated of, but the essays upon them are rather too concise. In a matter of this kind, the diagnosis and practical inferences require extreme and minute attention, and a treatise upon those affections should be more prolonged than the real extent of the subject would seem to require.

The fifth and last volume is nearly through the press, and will appear in a few weeks.

